# From Tutor to Future Educator: Investigating the Role of Peer-Peer Tutoring in Shaping Careers in Medical Education

**DOI:** 10.1007/s40670-024-02161-2

**Published:** 2024-09-09

**Authors:** Lauren Stokes, Harinder Singh

**Affiliations:** 1 Department of Medical Education, School of Medicine, University of California Irvine, Irvine, CA 92697 USA; 2 Department of Physiology & Biophysics, School of Medicine, University of California Irvine, Irvine, CA 92697 USA

**Keywords:** Peer tutoring, Medical students as teachers, Medical education, Future medical educators, Academic medicine

## Abstract

In the effort to promote academic excellence and provide teaching experiences and training for medical students, the University of California, Irvine, School of Medicine (UCISOM) built a novel peer tutoring program (2020), Collaborative Learning Communities with Medical Students as Teachers (CLC-MSAT). While the role of peer-assisted learning in student success on academic courses is well established, we wanted to assess the impact of our UCISOM peer-assisted learning program on tutors’ career interest in medical education. Through a mixed-methods analysis of our peer tutors’ experiences, we found 100% were overall satisfied with their positions; > 85% learned new skills; > 88% felt they were strong teachers; > 88% felt they now had a stronger grasp of the medical curriculum and most importantly, 100% of students maintained their interest and aspirations to serve in a future medical educator role after participating as a tutor. Additionally, we found a statistically significant relationship between tutors’ intentions of exploring a career in academic medicine after serving in the CLC program. Our findings suggest that our tutoring program has had a positive impact in providing authentic teaching experiences and training to medical students early in their careers at UCISOM, which may help prepare the next generation of qualified academic clinicians.

## Introduction

One of the main expectations of health professionals in an academic institution is teaching, yet there are often inadequate avenues for providing teaching training for medical educators [1]. To address the issue of a lack of training and increasing demand for medical educators, in recent years, medical education institutes have been involved in building peer-assisted learning (PAL) models. The intention is that these programs may provide avenues for medical students to serve as teachers while still pursuing medical education and possibly serve a role as medical educator while practicing medicine.

PAL refers to an education method in which a more senior-standing student provides instruction for their peers [2, 3]. The PAL models include the development and refinement of knowledge through active learning support from peers. PAL programs ensure student success and most importantly support the students who might be at risk of failing [4]. The strengths of PAL programs also include fostering a safe learning environment and providing mentorship opportunities for students [2]. Additionally, during the COVID-19 pandemic-induced virtual instructions and isolation, students expressed a preference for peer-peer learning over primarily relying on faculty instruction [5]. This momentum in innovating instruction and medical education can be captured to create teaching avenues for students.

Multiple studies have indicated that PAL programs have positive impacts for both tutors and tutees; for example, tutors were found to have significantly higher USMLE scores on Step 1 and Step 2 CK, as well as overall medical school GPAs [6, 7]. Additionally, some universities have found that for objective measures such as examination scores and course grades, students who participated in peer tutoring performed better, on average, than their non-participant peers [8–10]. For more subjective measures, such as collaboration and leadership skills, peer tutoring has been found to also have a positive influence on tutors [10–13]. Interestingly, it has been demonstrated that peer-led teaching not only aids in students becoming more receptive to teaching, but also develops an increasing interest for teaching in future years in medical school [9]. Strengths of PAL programs also include the prospect that peers are able to more accurately determine knowledge gaps compared to teacher-led learning, as well as creating a horizontal hierarchy in the teaching environment [14–16]. PAL programs are deemed as a bidirectional and reciprocal process in which the tutor naturally discovers their own level of content understanding based on their ability to teach the content to another person [17, 18]. This type of educational strategy is meant to supplement teacher-led learning as an additional resource to serve the wide learning levels of a student cohort [19, 20].

To fulfill the need of peer-peer learning and nurturing future generations of educators, in 2020, the University of California Irvine, School of Medicine (UCISOM) built a peer-assisted learning model to support students’ scholarly success during pre-clerkship and clinical years [5]. After a successful pilot program, MS1s were recruited to build a collaborative student community-based environment for the incoming class of MS1s. Services were tailored to successfully transition students into medical school, which included a focus on navigating the UCISOM landscape, relieving anxiety, managing COVID pandemic-induced loneliness, and learning medical physiology and anatomy in a low stress environment from their peers. This program was named the Collaborative Learning Communities with Medical Students As Teachers (CLC-MSAT) [21].

As the team grew from a staff of 18 to 73 peer tutors in a 4-year period (2020–2024), our attention has also turned into cultivating future academic-oriented clinicians interested in exploring medical education or academic medicine as a career. Specifically, our team is invested in providing timely, relevant, and ongoing professional development in evidence-based teaching pedagogy, diversity, equity, and inclusion (DEI) training, as well as mental health advocacy. For example, MSATs are required to participate in a suicide prevention certification training, referred to as question, persuade, refer (QPR), which helps MSATs determine the signs of students they may work with combatting mental health challenges. DEI workshops are led by experts at our university to ensure our programming remains inclusive of the diverse student body we are privileged to serve.

## Method

### Program Overview

CLC was initially developed through the collaboration between faculty, medical education administration, and medical students at UCISOM who identified the need and preference for peer-led instruction. The program continues to adapt to student and MSAT feedback with each iteration of the program, which takes place between July and May of each academic year. All MSATs are compensated equally and competitively. UCISOM MSATs receive nearly 60% higher compensation in comparison to baccalaureate-holding peer tutors at other medical school programs within the University of California system. All CLC services are provided at no-cost to students.

The CLC-MSAT sessions are divided into groups with one tutor per seven students, to allow enough bandwidth for a tutor to provide dedicated attention to all students in the group. Faculty members, including course directors, are assigned to each CLC-MSAT group to collaborate and provide teaching materials for in-house and board exams to the MSATs.

### Standards

UCISOM MSATs are compensated on an hourly basis and permitted to work up to 10 h per week. MSATs submit biweekly timesheets to the director of academic support, which documents the amount of time they spent on each employment activity. In addition to hours spent teaching, MSATs are also compensated for preparation time, administrative tasks such as organizing session schedules, and for leadership responsibilities, such as the distribution and analysis of feedback surveys. MSATs are considered academic employees at UCISOM, which entitles them to unique privileges, such as discounts to wellness, financial, parking, and leisure resources within the local community, which are typically reserved for full-time staff and faculty of the university. With each iteration of the program, we continue to attract the highest number of applications than in previous years. For example, in 2024, over 40% of medical students at UCISOM applied to serve as an MSAT.

### Service Offerings

**Fig. 1:**
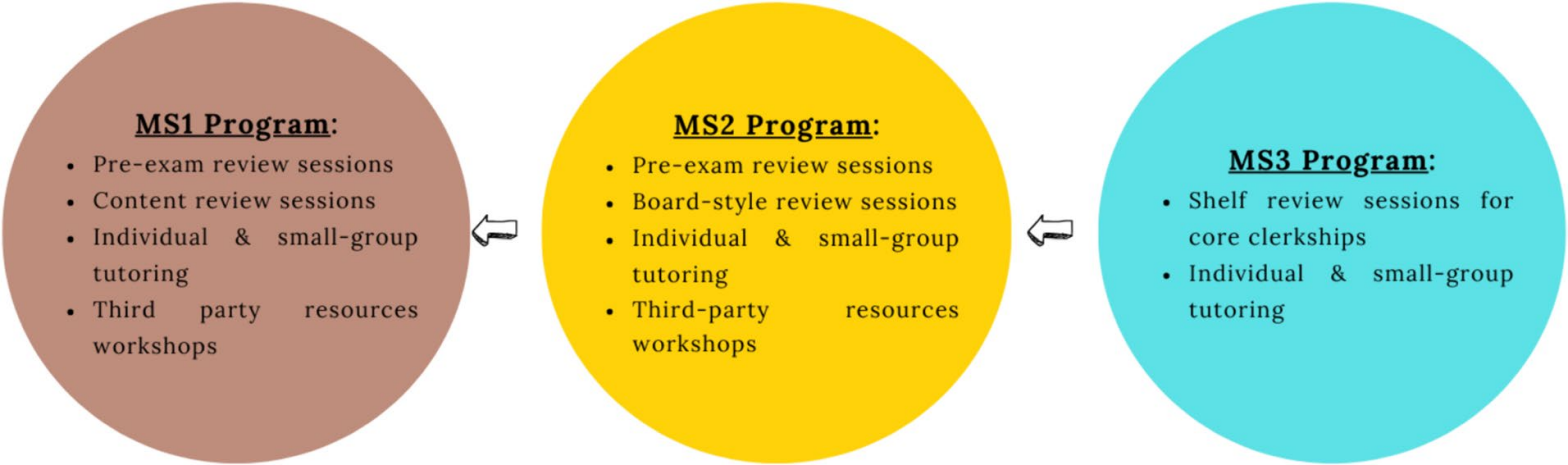
CLC-MSAT service offerings per program

Service offerings and number of MSATs differ by level of medical students served. For example, the MS1 program provides the most services and has the highest number of MSATs and highest student attendance across sessions. The three programs within CLC continue to expand in response to student and MSAT feedback. Details of the services offered to MS1s to MS3s (Fig. 1).

### MS1 Program

Second year medical students lead the CLC program for first year medical students. Services include pre-exam review sessions, content review sessions, individual and small group tutoring, and workshops related to utilizing third-party resources.

### MS2 Program

Third year medical students lead the CLC program for second year medical students. Services include pre-exam review sessions, board-style question sessions to help prepare students for Step 1, individual and small group tutoring, and workshops related to utilizing third-party resources.

### MS3 Program

Fourth year medical students lead the CLC program for third year medical students. Supplemental instruction is offered for each of the seven core clerkships, referred to at UCISOM as “rotation tracks.” Additionally, individual and small group tutoring are available to third year medical students.

### Leadership Organization and Structure

**Fig. 2:**
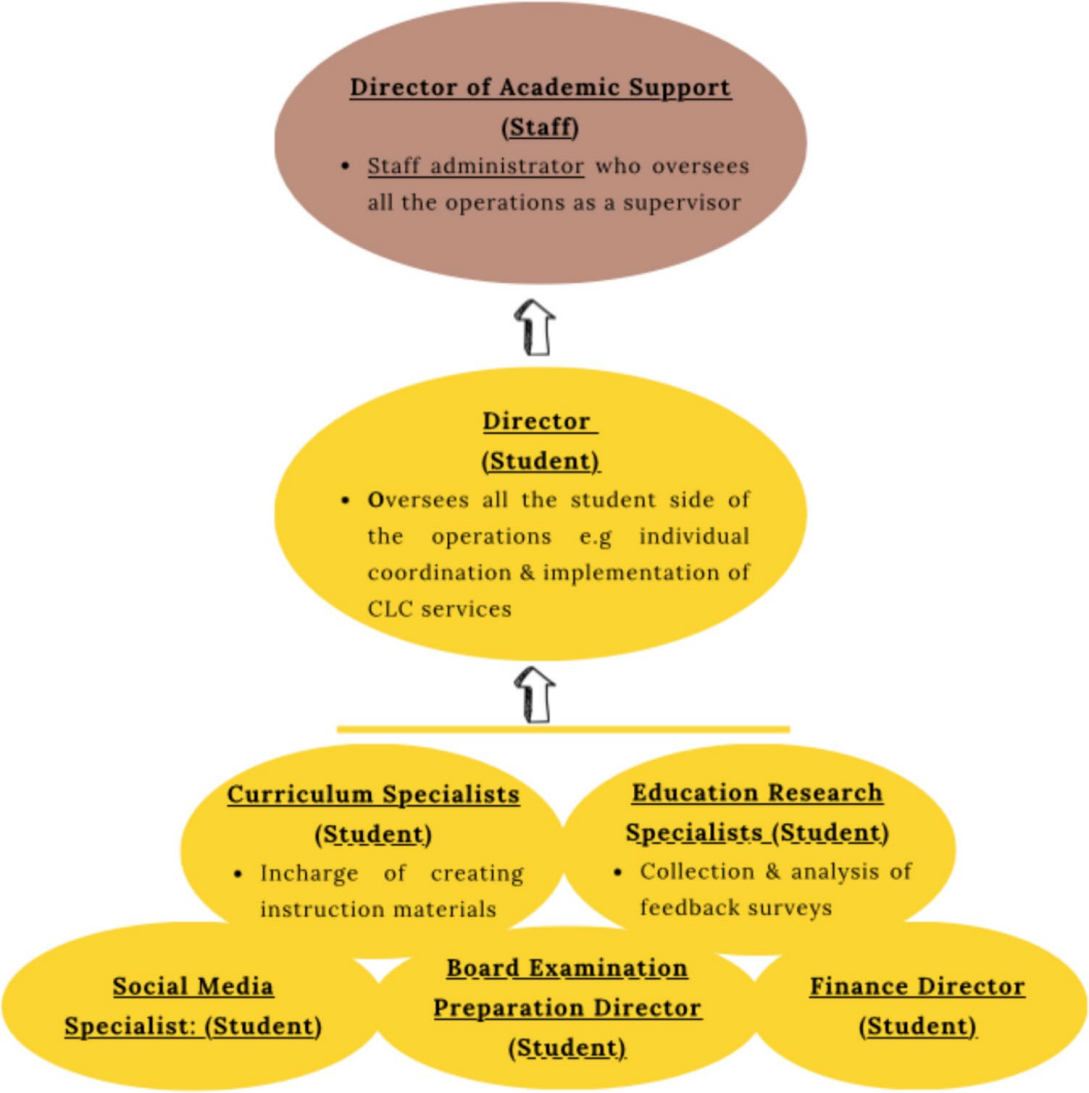
CLC-MSAT leadership organization and structure

The CLC-MSAT program is led by a staff administrator under the title of director of academic support services. This individual oversees the training of MSATs, implementation of tutoring services, and evaluation of the program. On the student leadership level, student directors oversee the individual coordination and implementation of CLC services. Additional student leadership positions include curriculum specialists, who are in charge of creating instructional materials, and education research specialists, who support the collection and analysis of feedback surveys. Throughout the past 3 years, additional leadership positions have emerged due to MSAT requests, such as a social media specialist, board examination preparation director, and finance director (Fig. 2). These positions are created to allow students an opportunity to gain specialized experience within CLC, with the intention of increasing the competitiveness of their curriculum vitae when applying for residency.

MSAT program directors meet with the faculty basic science and clinical science course directors at the beginning of each year for an introductory session about ways the program can support student learning and to glean insights into areas for improvement. Several faculty also opt to collaborate with our MSATs to host their exam reviews and/or share their teaching material to improve CLC-generated teaching worksheets.

### MSAT Selection Process

**Fig. 3:**
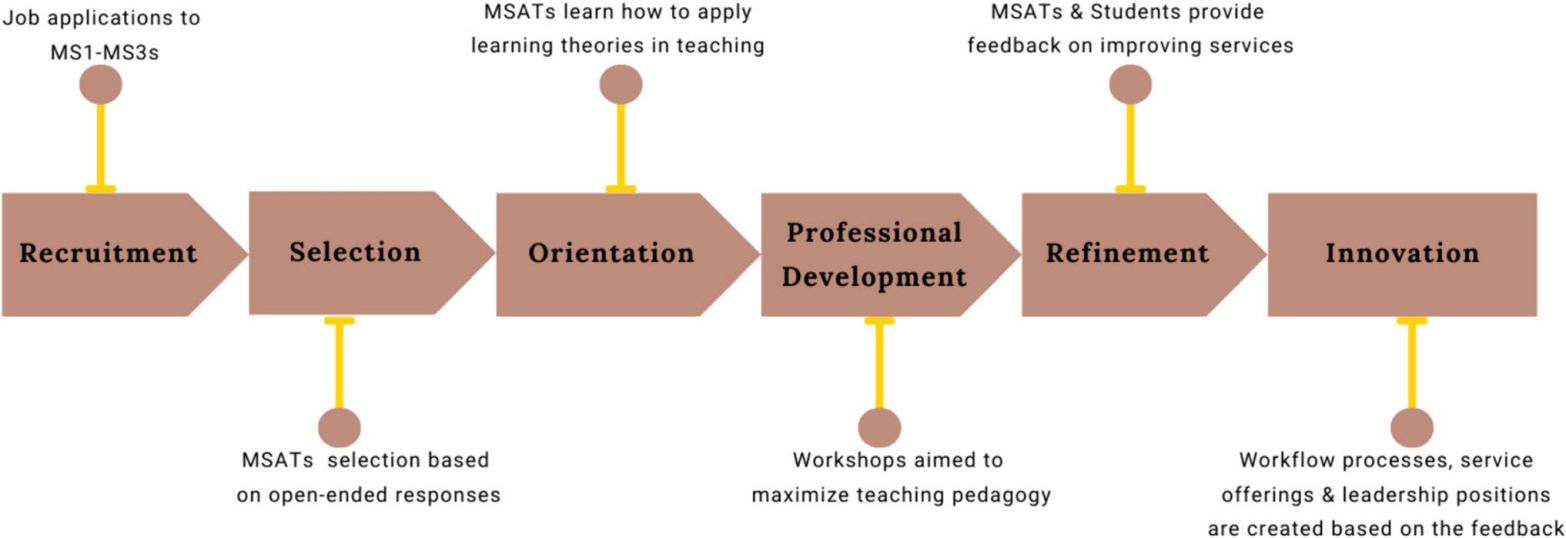
Annual timeline of CLC-MSAT program

CLC-MSAT recruitment takes place on an annual basis in which first through third year medical students can apply for a position in early Spring of each academic year (Fig. 3). While students must be in good academic standing to serve as an MSAT, there is no academic performance minimum, such as exam scores, to be considered for a position. Students are assessed holistically on their responses to open-ended questions using a standardized rubric. The review team consists of 35 current MSATs who score each application with all identifying information of the applicant removed. After the initial MSAT screening, the director of academic support unblinds all applications and reviews the applications with a minimum score of 45 out of 50. The number of applications received consistently exceeds the number of positions available, as over 40% of medical students apply for a position and about 50% of applicants are ultimately extended an offer of employment.

### Training and Professional Development

As a faculty trained in a secondary teaching credential program, the director of academic support services provides 5 h of initial training in teaching pedagogy and operations of the program. CLC training is rooted in social cognitive, adult, and situated learning theories. Specifically, our MSATs strive to support the reciprocal nature of peer-led learning by creating a safe environment for students of all learning levels, while focusing on content directly applicable to the practice of medicine. Additional areas of focus in training constitute creating actionable learning objectives, scaffolding, combatting impostor syndrome with students, presenting information in multiple modalities to target unique learning preferences, and harnessing participation strategies. Training is provided both in-person and via Zoom at the beginning of the academic year.

The focus of this paper is to provide an understanding of our MSATs’ experiences serving within the CLC-MSAT program, including their overall satisfaction, recommended areas for improvement, and exploring the potential impact of their involvement in the CLC-MSAT program has had on envisioning a future career in medical education.

## Data Collection

### Instrument

Our research team developed an inventory to help understand the impact that serving as an MSAT has had on our students, including how the experience has potentially impacted their career aspirations. Students who served as MSATs during the 2022–2023 academic year were invited to participate in the survey in May 2023. The survey questions included an informed consent to participate. A supplementary document with the survey questionnaire is attached with the manuscript, which provides an item requiring informed consent to participate. The inventory explored if the MSAT had prior teaching experience or formal teaching training before serving within the CLC program, satisfaction with their position, collaboration experiences with other MSATs and students, and how this role potentially had any impact on their career trajectory. The research project was deemed as exempt through the IRB process by the University of California, Irvine.

In addition to demographic information and previous teaching experience, the survey had a total of nine Likert scale items and three open-ended items to assess satisfaction, career trajectory, and areas for continued growth of the program. MSATs were invited to complete the survey by the UCISOM director of academic support services at the end of the third iteration of the program in 2023. All participation was voluntary and anonymous.

### Data Analysis Procedures

Quantitative survey items were analyzed using standard percentage calculations on Excel. The career trajectory responses were analyzed using SPSS (Statistical Package for Social Sciences) software to conduct a paired samples *t*-test. Qualitative items were independently analyzed in a three-step process. The first step involved the researchers independently determining a theme for each response to the open-ended items. In the second step, the researchers then further independently generated six overarching themes for the first open-ended item and five themes to combine the second and third questions. In the third step, the researchers compared their themes and categorized the data into final themes presented in the results.

### Previous Teaching Experience

In the effort to gauge previous teaching experience or formal training, MSATs were asked if their experience or training took place at the K-12, undergraduate, or graduate level, as well as “no previous experience or training.” A total of nine statements assessed satisfaction and career trajectory items that were based on a 1–4 Likert scale with a 1 indicating a strongly disagree response to the statement, whereas a 4 indicated a strong agreement. Single-question items included *Overall, I am satisfied with my position as an MSAT*, *I would recommend for other students to serve as an MSAT*, and *I have learned new skills in this position*.

To reflect on how the role has influenced their perceptions as a teacher, MSATs were asked: *after serving as an MSAT, I feel I am a stronger teacher*, as well as, *I have a stronger grasp of the medical school curriculum*. To address the notion of collaboration and effective teaching, two items included:

- *I work well with my colleagues in this program.*
- *I work well with my students in this program.*

To measure how the CLC program has potentially influenced the MSATs’ career trajectories, they were asked to rate:

- *Before I served as an MSAT, I envisioned a career in academic medicine.*
- *Now that I have served as an MSAT, I envision a career in academic medicine.*

### Open-Ended Items

In addition to the Likert scale items, three open-ended questions asked the following:

- *What are some of the aspects you enjoyed most about serving as an MSAT?*
- *Has serving as an MSAT had any influence on your career aspirations?*

Respondents were also provided with the opportunity to disclose areas for improvement in an item which posed:

- *How can the CLC-MSAT program improve to better support MSATs?*
- *Did you encounter any specific challenges as an MSAT?*

Finally, the third open-ended item requested for *any additional comments, concerns, or suggestions the MSAT wanted to share with the research team*.

### Demographic Items

MSATs were invited to disclose non-identifying demographic information, such as their current year and program in medical school, age, gender, and first or second college generation status. All demographic questions had the option of “prefer not to say” to ensure respondents did not feel any identifying information was being solicited.

## Results

### Demographics of Respondents

A total of 28 MSATs completed the MSAT Feedback Survey in May 2023 out of a team of 48 (response rate = 58%). In addition to serving in the current team, the academic years the respondents indicated serving as an MSAT were as follows: 2020–2021, 25% (*n* = 7); 2021–2022, 32% (*n* = 9); and 2022–2023, 100% (*n* = 28). Our total team consisted of 18 MS1 program MSATs, 15 MS2 program MSATs, and 15 MS3 program MSATs. All information presented in the figures below has been rounded up to the nearest whole number. Table 1 shows demographics data for all the CLC-MSAT program participants.

**Table 1:** Demographic information of MSAT respondents

Academic years as an MSAT	2020–2021 (*n* = 7)	2021–2022 (*n* = 9)	2022–2023 (*n* = 28)	
	25%	32%	100%	
Class standing	MS2 (*n* = 1)	MS3 (*n* = 11)	MS4 (*n* = 28)	Other (*n* = 2)
	4%	39%	43%	14%
Degree program	MD (*n* = 22)	MD/PhD (*n* = 3)	MD/MBA (*n* = 2)	MD/MPH (*n* = 1)
	78%	11%	7%	4%
Mission-based	Yes (*n* = 5)	No (*n* = 22)	Prefer not to say (*n* = 1)	
	18%	79%	3%	
Programs served as an MSAT (over multiple years)	MS1 (*n* = 20)	MS2 (*n* = 7)	MS3 (*n* = 9)	Prefer not to say (*n* = 1)
	71%	39%	39%	4%
Position(s) held by the respondents (over multiple years)	MSAT only (*n* = 19)	MSAT and director-level (*n* = 7)	MSAT and specialist/other (*n* = 10)	Prefer not to disclose additional leadership titles (*n* = 1)
	68%	25%	36%	4%
Age	22–25 (*n* = 8)	26–39 (*n* = 18)	30–33 (*n* = 2)	
	29%	64%	7%	
Gender	Male (*n* = 14)	Female (*n* = 14)		
	50%	50%		
College generation status	First generation (*n* = 1)	Second generation or greater (*n* = 27)		
	4%	96%		
Previous teaching experience	None (*n* = 3)	K-12 (*n* = 16)	Undergraduate (*n* = 20)	Graduate (*n* = 4)
	11%	59%	74%	15%
Previous teaching training	None (*n* = 13)	K-12 (*n* = 5)	Undergraduate (*n* = 11)	Graduate (*n* = 2)
	46%	18%	41%	11%

The breakdown of class standings of MSATs was 43% = MS4, 39% = MS3, 14% = “Other” (leave of absence or different educational track), and 3% = MS2. Most participants were MD-exclusive students (79%) compared to 11% of MD/PhD, 7% MD/MBA, and 4% MD/MPH. Additionally, 18% of respondents were students in one of the mission-based programs at UCISOM, which include LEAD PRIME-LC and PRIME LEAD-ABC compared to 79% of students not in a mission-based program. PRIME-LC trains future physicians to meet the needs of under-resourced Latino communities. PRIME LEAD-ABC produces physician leaders who address the diverse health needs of Black communities. One student indicated “prefer not to say” regarding their mission-based program status.

Since the inception of CLC in 2020, several upper class-standing students have served in multiple CLC-level programs over the past 3 years. Most participants (71%) served in the MS1 program, compared to 39% in the MS2 program, 39% in the MS3 program, and 4% who preferred not to disclose which program they had ever served as an MSAT. While 68% of respondents served as an MSAT-only (*n* = 19), 25% held a student director level role (*n* = 7), 25% held a specialist position (*n* = 7), 11% indicated an “Other” position (*n* = 3), and 4% preferred not to say (*n* = 1).

Based on age range, 64% of MSATs indicated they were between 26 and 29 years old, 29% were between 22 and 25 years old, and 7% were between 30 and 33 years old. Exactly 50% of the participants identified as male (*n* = 14) compared to 50% who identified as female (*n* = 14). Based on college generation status, 96% of MSATs were a second-generation college student compared to 4% who were the first in their immediate family to attend college.

### Professional Preparation

Based on prior teaching experience, 74% of MSATs had served in a teaching capacity at the undergraduate level (*n* = 20), 59% at the K-12 level (*n* = 16), 15% in graduate school (*n* = 4), and 11% of the MSATs had no prior teaching experience before the CLC-MSAT program (*n* = 3). Nearly half of the MSATs had no formal teaching training (48%) compared to 19% to teach at the K-12 level, 41% for undergraduate students, and 11% for graduate students.

### MSAT Satisfaction

MSAT satisfaction was measured on a 1–4 Likert scale with a 1 indicating a strong disagreement and a 4 indicating a strong agreement. When asked to rate their overall satisfaction as an MSAT, 68% (*n* = 19) indicated they strongly agreed they were satisfied compared to 32% (*n* = 9) who agreed to the same statement. No MSATs indicated a disagreement or strong disagreement regarding their satisfaction.

A total of 76% of MSATs strongly agreed (71%) or agreed (25%) they would recommend for other students to serve as an MSAT compared to one student (4%) who indicated they disagreed with this statement.

### Personal Development

To measure personal and professional development, 86% of respondents strongly agreed (50%) or agreed (36%) they learned new skills in the position compared to 14% who disagreed. Most MSATs (90%) strongly agreed (61%) or agreed (29%) they were a stronger teacher after serving as an MSAT compared to 10% who disagreed. An item to measure if MSATs gained a stronger grasp of the medical curriculum as a result of serving as an MSAT, 90% strongly agreed or agreed to the statement compared to 10% who disagreed.

### Collaboration Effectiveness

MSATs were asked to gauge their effectiveness in collaborating with their colleagues and students in the program. Most MSATs (97%) strongly agreed (78%) or agreed (19%) they worked well with their colleagues compared to 3% who disagreed. In comparison, 29% of MSATs strongly agreed or agreed (64%) they worked well with their students.

### Career Trajectory

**Fig. 4:**
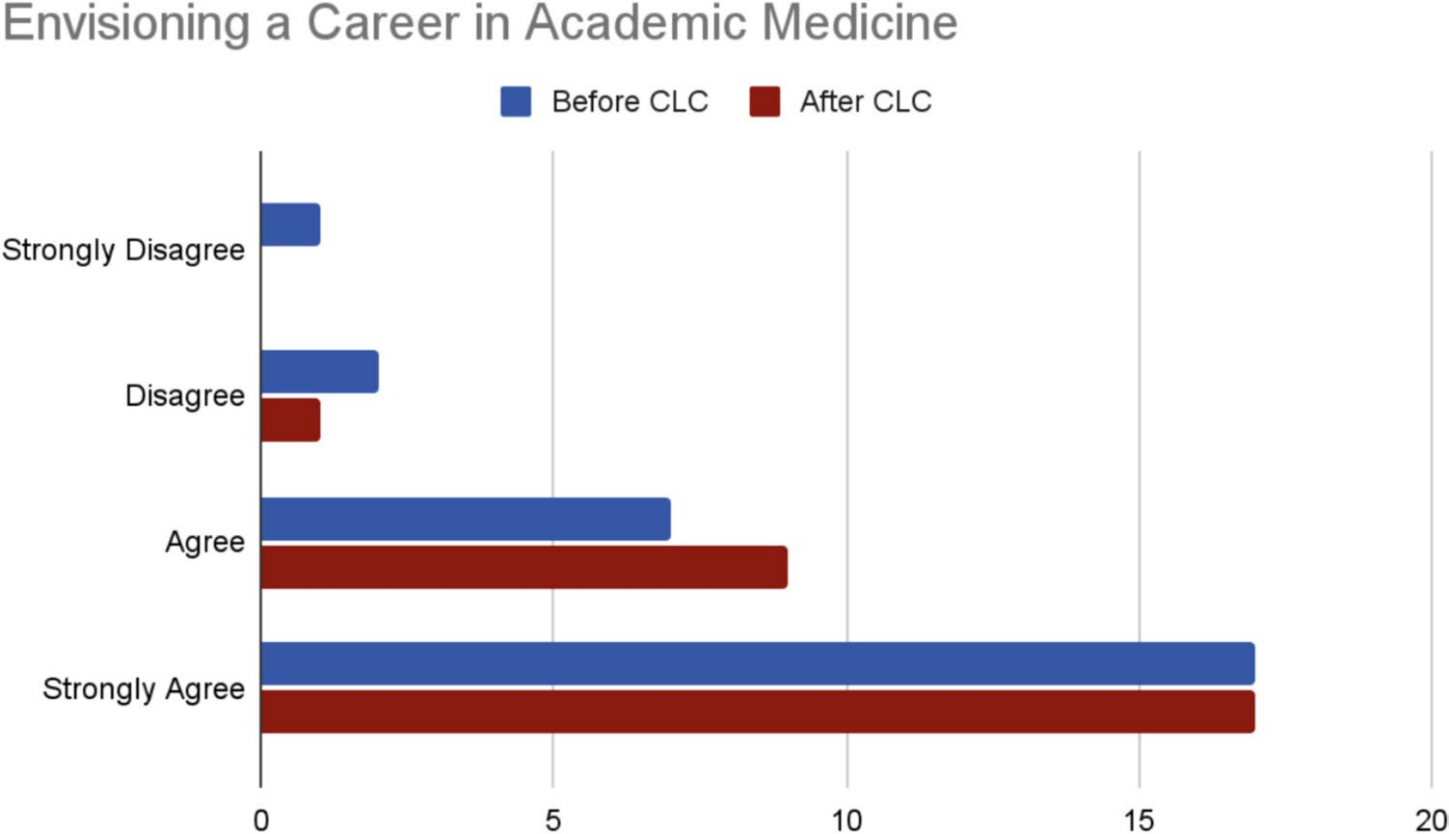
Interest in a career in academic medicine before and after serving as an MSAT

A paired samples *t*-test was performed to measure the influence serving as an MSAT has had on their intended career trajectory in academic medicine. There was a statistically significant difference in agreement levels for the interest of pursuing a career in academic medicine before serving as an MSAT (*M* = 3.48, *SD* = 0.80) compared to after serving as an MSAT (*M* = 3.59, *SD* = 0.57); *t*(26) =  − 1.14, *p* < 0.001 (Fig. 4). While most MSATS (90%) strongly agreed (61%) or agreed (29%), about 10% of the team disagreed (7%) or strongly disagreed (3%) that they envisioned a career in academic medicine before serving as an MSAT. After serving as an MSAT, 96% of the team either strongly agreed (63%) or agreed (33%) they envisioned a career in academic medicine compared to 4% who disagreed. No respondents strongly disagreed with this statement. One participant’s response was removed from the results, as they did not provide both a before and after score in their response.

### Open-Ended Items

Three open-ended items invited MSATs to disclose their thoughts about the specific aspects they enjoyed while serving in CLC, as well as how this opportunity has potentially impacted their career trajectory. Recommendations for program improvement were solicited with an additional platform to disclose any remaining comments and concerns. General themes are presented below.

**Fig. 5:**
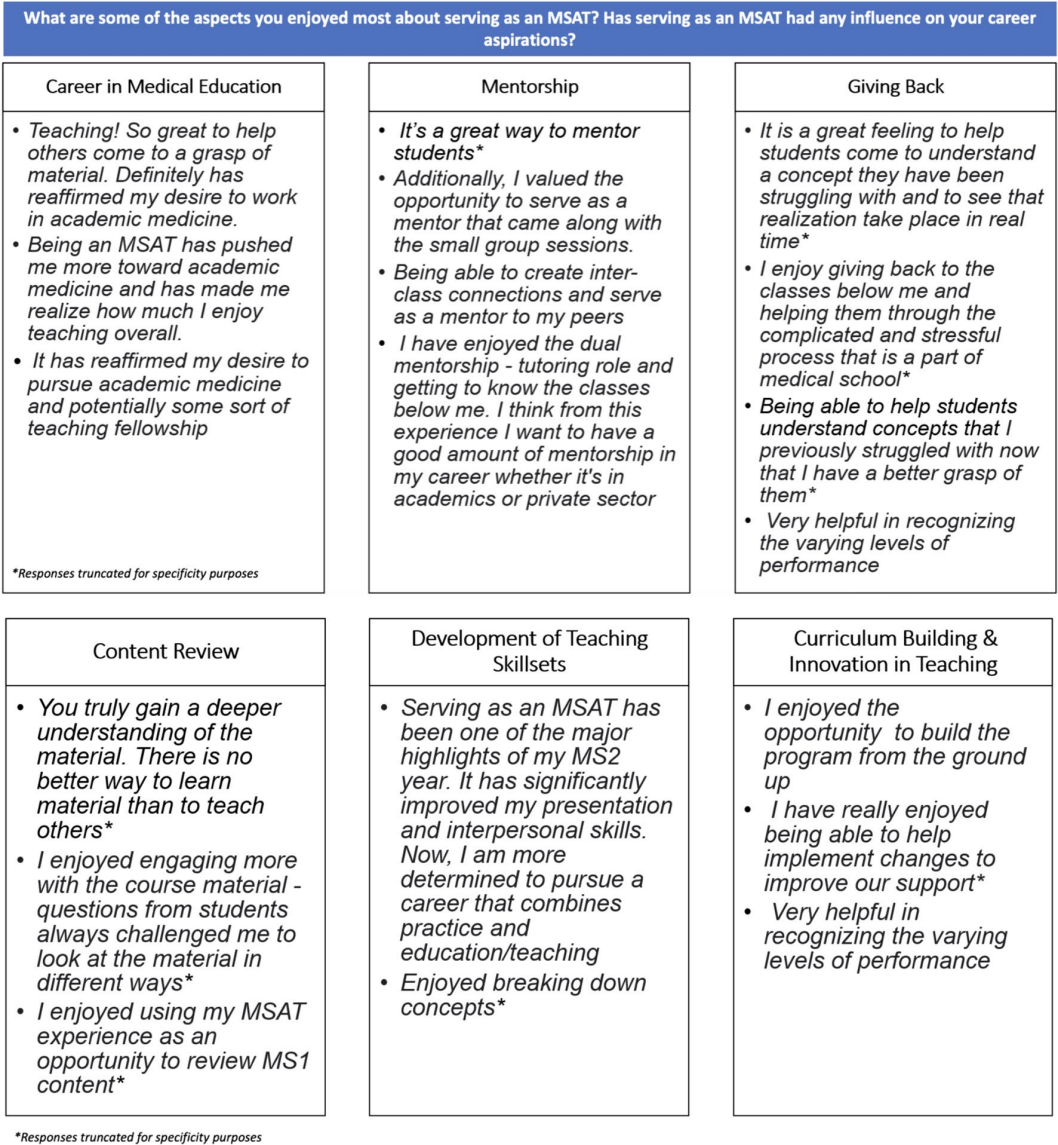
Aspects MSATs enjoyed and influences on career aspirations

The first open-ended item invited MSATs to share the aspects they enjoyed most about serving in the program (Fig. 5). In addition, MSATs were asked to discuss if the program had any influence on their career aspirations in academic medicine. A total of 17 MSATs (61% of the sample) provided responses to this item with most respondents sharing multiple ways CLC has impacted their career aspirations. Thus, response frequencies are reported in the number of responses per theme in lieu of a percentage of responses. Themes included a commitment to exploring a career in medical education (5 responses), enjoyment in mentorship and giving back (15 responses), engaging in content review (4 responses), developing teaching skill sets (3 responses), and participating in curricula building and teaching innovation (2 responses).

**Fig. 6:**
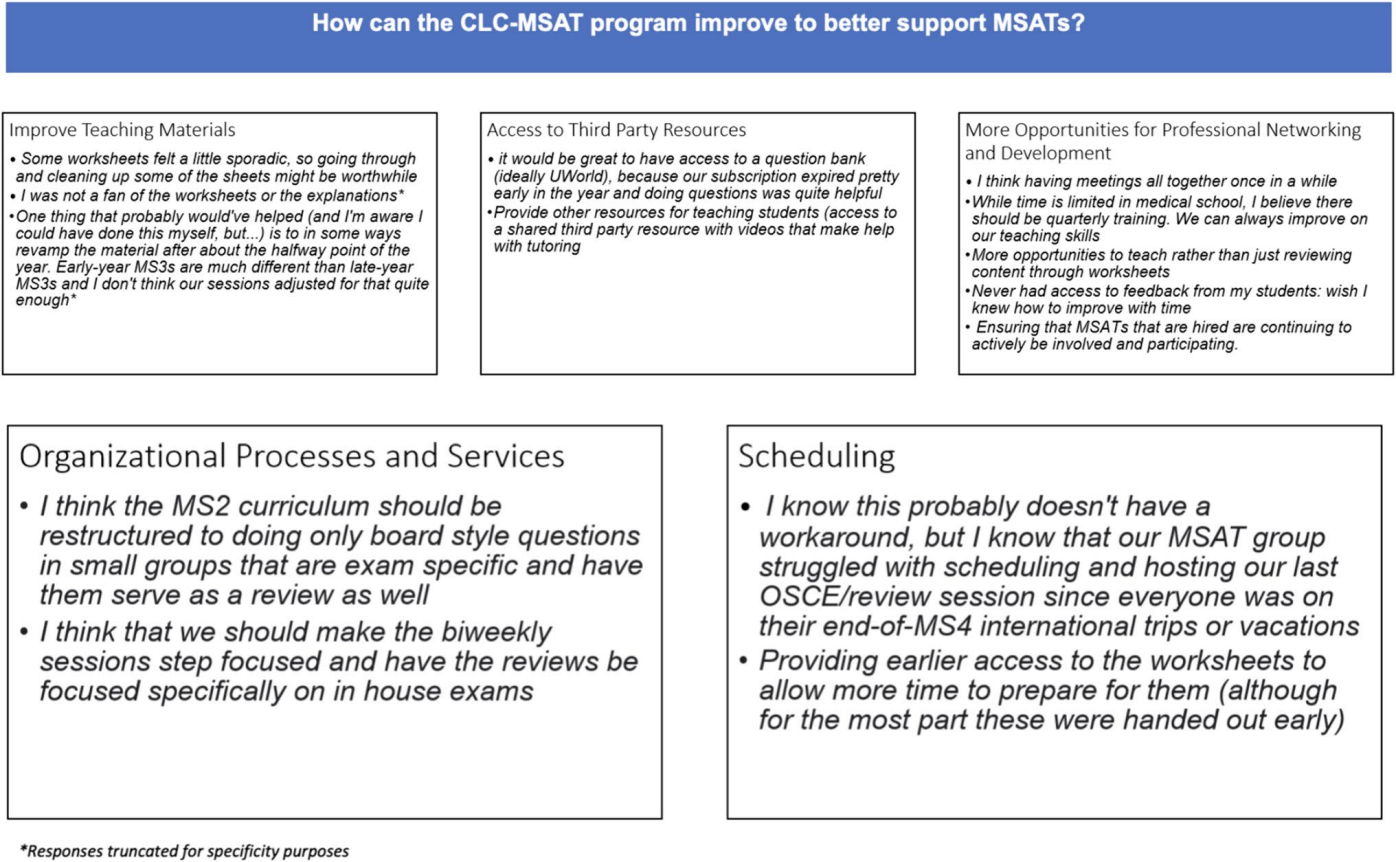
Improvements and general comments from MSATs

The second open-ended item invited MSATs to discuss ways CLC can improve to better serve student staff. A total of 15 MSATs (54% of the sample) provided responses to this item. Due to the similarities in the two open-ended items, the third and final open-ended item invited MSATs to share any additional comments or suggestions not captured in the other questions (Fig. 6). A total of 3 MSATs (11% of the sample) provided responses to this item, with most respondents sharing multiple areas that could be improved. Themes included improving teaching materials (3 responses), providing access to third-party resources for MSATs (2 responses), increasing the number of opportunities for professional networking and development (4 responses), improving organizational processes and services (6 responses), and addressing scheduling conflicts (6 responses).

## Discussion

The CLC-MSAT program at UCISOM has been designed to both promote academic excellence for the students in our medical program and to provide authentic teaching experiences for our future physicians. Of particular interest for the researchers was to determine if serving as an MSAT has had any influence on our UCISOM students’ career aspirations into academic medicine, as well as to determine areas of success and improvement for our program.

Based on our survey results, while 11% of MSATs had no previous teaching experience before CLC, 46% also had no previous teacher training (Table 1). All MSATs indicated they agreed or strongly agreed they were satisfied with their position, overall. Within these respondents, 96% of MSATs agreed or strongly agreed they would recommend this role to others. Additionally, 90% agreed or strongly agreed that serving as an MSAT has provided them with a stronger grasp of the medical curriculum and made them a stronger teacher (Table 2). As a theme of teaching efficacy, 93% of MSATs agreed or strongly agreed they worked well with their students.

**Table 2:** Satisfaction, professional development, and effectiveness of MSAT program to respondents

	Strongly agree	Agree	Disagree	Strongly disagree
Satisfied with position	68%	32%	0%	0%
Recommend others to serve as MSAT	71%	25%	4%	0%
Learned new skills	50%	36%	14%	0%
Stronger teacher after being MSAT	61%	29%	10%	0%
Stronger grasp of medical curriculum	79%	11%	10%	0%
Work well with colleagues	78%	19%	3%	0%
Work well with students	29%	64%	7%	0%

Our interest was also to understand if serving as an MSAT had any influence on a career in academic medicine. These findings indicated 89% of MSATs agreed or strongly agreed they envisioned this career before serving as an MSAT compared to 96% who agreed or strongly agreed after serving as an MSAT. Interestingly, one MSAT indicated they strongly disagreed that they envisioned a career in academic medicine before CLC; this statistic dropped to 0% of MSATs disagreeing or strongly disagreeing they wanted to be in academic medicine after serving in CLC. This is a worthwhile result to note, as the demands of serving as an MSAT did not lead to any attrition for our students in their commitment to a future career of teaching and medicine.

As we focus on the experiences of our MSATs within the CLC program, it is essential to examine the unique characteristics of our respondents. For example, the 2022–2023 MS1 program was staffed by 18 MSATs, the MS2 program had 14 MSATs, and the MS3 program had 15 MSATs. Within our particular sample, 82% of respondents served in either the MS2 or MS3 programs despite accounting for only 62% of the overall number of MSATs in the program. Thus, the majority of our responses were derived from respondents in the clerkship phase of their medical school journey.

This is an important distinction to highlight, as students in the latter phase of their medical school journey may have more concrete career plans than students in their pre-clerkship years. As such, these upper class-standing students may have joined the CLC program with clearer intentions as to how this program could facilitate their career aspirations compared to pre-clerkship MSATs who may still be in an exploratory phase of their post-graduation plans.

Additionally, 61% of respondents indicated they held a student leadership role in CLC, which would constitute a director or specialist title. A total of 18 students, or 40% of MSATs, held a leadership title for this iteration of CLC. The high representation of student leaders in these responses may have skewed the overall representation of MSATs’ experiences. Essentially, the MSATs who sought and executed leadership roles tended to be students who vocalized their desire to pursue academic medicine after CLC, which was a strong factor as to why their application was originally accepted for the position.

In lieu of these considerations, we are pleased to see a high overall satisfaction rate for our MSATs in addition to sentiments that serving in this program has refined or confirmed many of their aspirations to pursue a career in academic medicine. This is an important area to highlight, as peer tutoring programs such as CLC can help identify and capture students who are interested in academic medicine at an early point in their career. Providing these future educators with opportunities to practice evidence-based teaching pedagogy with students may help sustain a continued investment in pursuing a career which combines teaching and medicine.

## Strengths and Positive Outcomes

### Career in Medical Education

Before serving in the CLC program, most MSATS (89%) strongly agreed (61%) or agreed (29%) they envisioned a career in academic medicine. However, after serving as an MSAT, 96% of the team either strongly agreed (63%) or agreed (33%) they envisioned a career in academic medicine. Part of the modest 7% increase could potentially be explained by the naturally self-selecting bias of our sample; most MSATs held interests in academic medicine even before CLC employment, which likely attracted them to join the program in the initial phase.

Open-ended responses within the impact CLC has had on career aspirations included the enjoyment MSATs found in helping students grasp difficult content, which reaffirmed their desire to explore academic medicine. The personal, perceived teaching efficacy of helping students master content they once struggled with may has potentially contributed to MSATs’ perspective of being an effective teacher.

### Mentorship

The primary currency for the successful functioning of medicine and medical education is a healthy mentor–mentee relationship. The significance of mentorship in education has been emphasized at multiple levels. It was encouraging to see that MSATs also perceived this as a great opportunity to mentor the next generation.

### Giving Back

Related to teaching efficacy and mentorship attributes included the theme of giving back. Specifically, MSATs developed strong positive associations with assisting struggling students and seeing their development and confidence transform in real-time. MSATs also observed and learned how to recognize students at varying levels of performance to help foster teaching pedagogy to meet the students where they were currently at in their learning.

### Content Review

A natural benefit to the tutor-tutee relationship is for the tutor to gain a deeper understanding of challenging material in the effort to teach it to another student. MSATs reported using their position as an opportunity to review previous material and to master the content in a way they had not been able to previously as a student. Additionally, student questions allowed for MSATs to view material under different lenses, further strengthening their grasp of medical content.

### Development of Teaching Skill Sets

CLC was reported to be the highlight of one of the MSAT’s second year of medical school. Specifically, one respondent indicated the program helped improve their presentation and interpersonal skills. Combined, these factors motivated the MSAT to pursue a career that combines medical practice with education and teaching.

### Curriculum Building and Innovation in Teaching

The opportunities for innovating and improving the CLC program were reported as highlights for several MSATs. For example, MSATs discussed how they were able to help build the program “from the ground up” and participated in implementing changes to improve service offerings. MSATs take an active role in executing and improving CLC, which was meaningful for them to recognize.

## Feedback and Improvements

The program has been a great success at UCISOM and has helped both the tutors and tutees in mastering essential medical concepts and competencies. While the program has had significant positive outcomes, the overall interpretation on the impact of this program for MSAT involvement in future pursuits in medical education requires longitudinal tracking and alumni data collection. Therefore, the current data only shows the impact of this program on medical education trajectories of medical students at UCISOM. The assessment of the impact of peer tutoring programs in advancing medical education at national level warrants further exploration.

As evidenced by our ongoing surveys, our program is invested in supporting our MSATs by using their feedback to inform our services. Each of these items is outlined below based on the qualitative data captured on our survey.

### Improve Teaching Materials

Each year of CLC builds upon materials from the previous year. Our materials consist of worksheets with board-style questions that are derived from influences of existing resources (practice question banks) and in-house vignettes co-designed by our faculty. A new process addition to help improve our teaching materials is for the student curriculum specialists to send their materials to the staff director of CLC, as well as to the faculty course director of the particular content area. While the faculty course director assesses content accuracy and high yield appropriateness, the staff director makes recommendations on learning objectives, organization, and scaffolding opportunities to meet diverse learning levels of students. Additionally, most CLC materials pass through a review of at least five MSATs for proofreading.

### Provide Access to Third-Party Resources

In 2023, UCISOM provided free vouchers to the practice question bank, UWorld, to all second year medical students. Due to the costs associated with this offering, it is currently unclear if the university will be able to provide the same access to third-party resources for all levels of medical students. This is an area of consideration which will be revisited with each fiscal year.

### Increase Opportunities for Professional Networking and Development

In response to requests for more opportunities for professional networking and development, the staff director of CLC-MSAT instituted the following changes and mandatory trainings for all MSATs: introduced diversity, equity, and inclusion (DEI) sessions, mental health workshops, as well as improved initial and ongoing teaching pedagogy instruction.

Two DEI trainings are hosted by DEI experts within the UCISOM and UCI main campus departments. The sessions focus on implicit bias, cultural competency, identity salience, and intersecting identities as they relate to strategies and techniques for effective cross-cultural mentoring and advising. Mental health workshops are hosted by the UCI Counseling Center, which includes a suicidal ideation certification program for our MSATs, referred to as question, persuade, refer (QPR), to identify students at risk for mental health challenges. The initial and robust teaching training took place before the start of the 2023–2024 CLC-MSAT program, which was hosted by the staff director of CLC, who is also an adjunct STEM professor and received teaching credential training for secondary education. Additional pedagogy training is hosted in conjunction with UCI’s Division for Teaching and Educational Innovation department to help MSATs adopt and refine their skills for common teaching challenges, such as increasing student participation.

In addition, the number of staff meetings has increased for the 2023–2024 CLC-MSAT program in which weekly, biweekly, and monthly meetings are held with specific MSATs on the team. For example, weekly meetings are held between the student program directors and the staff director of CLC, biweekly for MSATs in specialist roles, and monthly for all other MSATs.

### Improve Organizational Processes and Services

To address areas of improvement in organizational processes and services, our CLC team has been increasingly explicit in communication regarding CLC positions, time commitments, and expectations. Although we have operated using position descriptions from the pilot year of our program, we have made previously ambiguous information more concrete. For example, we have expanded language from, “the MS1 education research specialist will conduct surveys throughout the year,” to “the MS1 education research specialist will be responsible for revising, distributing, analyzing, and reporting data for the MS1 program every October, December, and February of the academic year.”

In addition, CLC leadership has been more intentional about conveying expectations by role, as individual tutoring sessions have notoriously been the most difficult service to staff. As such, MSATs must confirm their acceptance that they will be expected to serve as a 1:1 tutor in addition to their large group teaching sessions when formally accepting their employment offer. This one change has led to simpler staffing processes for our individual tutoring sessions compared to previous years.

Another improvement area of organizational processes relates to navigating interpersonal differences between MSATs. For example, although an MSAT may share a leadership title of director or specialist, they still remain in a horizontal reporting structure to other MSATs. To help our team navigate challenges they may inevitably face with one another, we have increased the frequency and transparency of our meetings, as our quality communication channels have helped mitigate previous communication challenges. The CLC-MSAT team remains a united front, committed to providing robust and inclusive tutoring resources to those we are privileged to serve.

While we strongly believe that a peer tutoring program such as CLC provides an invaluable service to students and tutors, we recognize the unique constraints of individual institutional budgets, student support services (DEI, wellness, etc.), and staffing must be considered for this investment.

## Future Directions

Our CLC-MSAT program has been synonymous with seeking feedback and adapting to change with timely and relevant support for our MSAT team. While we have introduced new and robust trainings for our team, we are also continuously creating new leadership positions for our MSATs. Our intention is to help our MSATs gain valuable experience in a setting which supports and competitively compensates their contributions. For example, we have created director and specialist-level roles based on MSAT requests, including finance, social media, and board exam responsibilities. Our team regularly conducts research and presents and publishes on our findings to provide ample opportunities for MSATs to gain this invaluable experience.

Future directions for CLC-MSAT include longitudinal research to track where our graduated MSATs find their careers after residency. We are excited to likely be able to determine this data for several of our MSATs from our pilot program in the next 2 years. Additional areas for CLC include increased marketing of our services to undergraduate students interested in UCISOM to showcase the variety of careers in medicine and the doors that may open as a result of serving as an MSAT.

In order to provide additional pedagogy training to MSATs, we plan to sponsor interested MSATs to attend the annual conference on Educating Learners through Innovation and Technology (ELITe) organized by the Medical Education unit of University of California Irvine School of Medicine. During this 2-day conference, the instructors teach principles of effective learning through innovations in instructional technology, curriculum development and design, evidence-based slide design, writing effective learning objectives, gamification of medical education, building DEI and cultural competency into curricula, formative and summative evaluations, and program evaluations.

Historically, this conference has been attended by residents, fellows, and other physicians interested in medical education. Recently, many of the basic science faculty who are teaching pre-clerkship curriculum are also taking advantage of this training. Our plan is to open fee waivers for interested MSATs who would like to further advance and refine their teaching in medical education skills. We hope that this training will help fill the current gap of medical educators in the field.

## Conclusion

As evidenced by CLC’s continued expansion from a team of 18 MSATs in 2020 to 73 MSATs 3 years later, our attention is equally invested in supporting academic excellence for our students in addition to cultivating skilled, future academic clinicians. We continuously seek feedback from our MSATs to support their career trajectories. Results from this feedback include providing new service offerings for our staff, such as DEI, mental health certifications, and initial and ongoing teaching pedagogy. We are responsive to our MSATs’ needs and committed to providing any support which may help contribute to our MSATs pursuing careers in academic medicine.

## Data Availability

The data used to support the findings of this study are available here.
